# Fire Performance of FRP-Composites and Strengthened Concrete Structures: A State-of-the-Art Review

**DOI:** 10.3390/polym18020181

**Published:** 2026-01-09

**Authors:** Junhao Zhou, Yingwu Zhou, Menghuan Guo, Sheng Xiang

**Affiliations:** 1 Guangdong Provincial Key Laboratory of Durability for Marine Civil Engineering, Shenzhen University, Shenzhen 518060, China; 2 School of Mechanics and Construction Engineering, Jinan University, Guangzhou 510632, China

**Keywords:** fiber-reinforced polymer, fire performance, FRP-reinforced concrete

## Abstract

The structural application of Fiber-Reinforced Polymers (FRP) is significantly hindered by their inherent thermal sensitivity. This paper presents a comprehensive review of the fire performance of FRP materials and FRP-concrete systems, spanning from material-scale degradation to structural-scale response. Distinct from previous studies, this review explicitly distinguishes between the fire behavior of internally reinforced FRP-reinforced concrete members and externally applied systems, including Externally Bonded Reinforcement (EBR) and Near-Surface Mounted (NSM) techniques. The thermal and mechanical degradation mechanisms of FRP constituents—specifically reinforcing fibers and polymer matrices—are first analyzed, with a focused discussion on the critical role of the glass transition temperature T_g_. A detailed comparative analysis of the pros and cons of organic (epoxy-based) and inorganic (cementitious) binders is provided, elaborating on their respective bonding mechanisms and thermal stability under fire conditions. Furthermore, the effectiveness of various fire-protection strategies, such as external insulation systems, is evaluated. Synthesis of existing research indicates that while insulation thickness remains the dominant factor governing the fire survival time of EBR/NSM systems, the irreversible thermal degradation of polymer matrices poses a primary challenge for the post-fire recovery of FRP-reinforced structures. This review identifies critical research gaps and provides practical insights for the fire-safe design of FRP-concrete composite structures.

## 1. Introduction

Fire remains one of the most destructive hazards to human life and the built environment. According to the China Fire and Rescue Bureau, fire and rescue teams nationwide responded to approximately 908,000 fire incidents in 2024, resulting in 2001 deaths and direct economic losses of about 7.74 billion yuan. Building fires accounted for more than 40% of these events, with incidents in high-rise residential towers, underground transport systems and critical public infrastructure exhibiting a persistent upward trend. In addition to immediate casualties and direct property damage, fire disasters frequently trigger secondary consequences—including structural collapse, smoke inhalation injuries, secondary explosions and large-scale environmental contamination—that amplify their societal and economic impact. At the global scale, the International Association of Fire and Rescue Services (CTIF) World Fire Statistics report on the order of tens of millions of fire incidents annually (e.g., about 32 million incidents and 16,900 deaths in 2017), underscoring that fire safety is a systemic challenge rather than a sequence of isolated accidents.

Recent catastrophic events have further highlighted the vulnerability of densely populated high-rise developments, especially where combustible façade or renovation materials are involved. A striking example is the 2025 Wang Fuk Court apartment fire in Tai Po, Hong Kong, which rapidly evolved into the city’s deadliest blaze since 1948 [1]. The fire, which started on bamboo scaffolding and flammable renovation materials on the exterior of a 32-storey block, spread along polystyrene foam panels, construction mesh and other polymer-based products to engulf seven of the estate’s eight towers. Despite the deployment of roughly 2300 firefighters and more than 200 fire engines, it took over 40 h to fully extinguish the fire; at least 128 people were killed, around 70–80 people were injured and more than 150 people remained unaccounted for in the immediate aftermath. Subsequent investigations pointed to a combination of highly combustible external materials, ineffective or non-operational fire alarm systems and inadequate site management and regulatory enforcement as key factors that allowed a localized façade fire to escalate into an estate-wide catastrophe. Together with earlier events such as the 2010 Jing’an District high-rise fire in Shanghai [2], which caused 58 fatalities and was also linked to combustible exterior insulation and renovation materials, these incidents demonstrate that modern urban fires are increasingly governed by the behavior of polymer-rich, lightweight construction and retrofit systems rather than traditional concrete and steel alone.

With the increasing integration of advanced materials into modern construction, transportation, water management, and energy systems, fiber-reinforced polymer (FRP) composites have emerged as a critical class of structural materials in civil engineering. Their high specific strength, low density, excellent corrosion resistance, and ease of fabrication have enabled their widespread use in structural strengthening, FRP-reinforced concrete systems, modular FRP profiles, rail transit components, and marine infrastructure [3,4,5]. Based on fiber type, FRP composites are commonly grouped into carbon FRP (CFRP), glass FRP (GFRP), aramid FRP (AFRP), and basalt FRP (BFRP) [6,7], each offering distinct mechanical and thermal performance characteristics (Table 1). This review primarily concentrates on the civil engineering applications of CFRP and GFRP, which dominate current practice.

**Figure 1:**
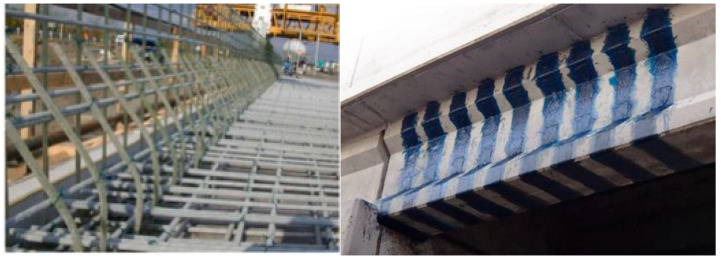
The application of FRP in civil engineering [17,18].

FRP composites are utilized in two principal forms: (1) externally bonded reinforcement for strengthening and retrofitting existing structures, and (2) internal reinforcement through FRP bars, tendons, or tubular profiles that partially or fully replace traditional steel [6,9,10,11,12,13,14,15,16]. As external strengthening materials, FRP systems provide several advantages over steel plates, including superior strength-to-weight ratios, minimal added dead load, inherent corrosion resistance, and straightforward installation, making them particularly suitable for upgrading beams, columns, slabs, and bridge elements subjected to heightened service demands or deterioration [6,9,10,11,12,16]. When used as internal reinforcement or structural components, FRP enhances durability by mitigating chloride ingress, moisture penetration, and chemical degradation, ultimately extending the service life of concrete structures [9,13,14]. Figure 1 shows common practical applications of FRP.

Despite these advantages, the inherently poor fire performance of FRP composites remains a primary barrier to their broader structural application. In typical compartment-fire scenarios, temperatures can reach 300–400 °C within 3–5 min and exceed 800 °C within 10 min. Under such extreme thermal exposure, FRP composites experience rapid degradation due to polymer matrix softening, decomposition, and loss of fiber–matrix interfacial bonding, resulting in severe reductions in mechanical capacity and, in extreme cases, ignition or toxic fume release [19,20].

As FRP use continues to expand across civil infrastructure, improving the fire resistance of FRP composites and FRP–concrete systems has become an urgent research imperative. This review provides a comprehensive examination of the thermal and mechanical behavior of FRP materials at elevated temperatures, the fire performance of FRP-strengthened and FRP-reinforced concrete structures, and the underlying mechanisms governing their degradation. By synthesizing current state-of-the-art knowledge, this review aims to support the development of safer FRP-based structural systems and inform the refinement of future fire-design guidelines.

## 2. Thermal and Mechanical Degradation of FRP Components

It should be mentioned that the construction industry has become the largest consumer of FRP composites in recent years. The production and application of FRP materials are inseparable from the resin, which acts as a load transfer or adhesive. The mechanical properties of FRP composites will degrade significantly with the rising of temperatures, which is attributed to the characteristic of fibers and polymer matrix. Among various disasters, fire is considered a high-frequency and destructive disaster, causing massive casualties and huge property losses every year. So, it is crucial to understand the change of FRP composite at elevated temperature. This section provides a review and discussion about the fire behavior of fibers, organic matrix, and FRP-concrete members.

### 2.1. Mechanical Properties of Fiber at Elevated Temperature

**Figure 2:**
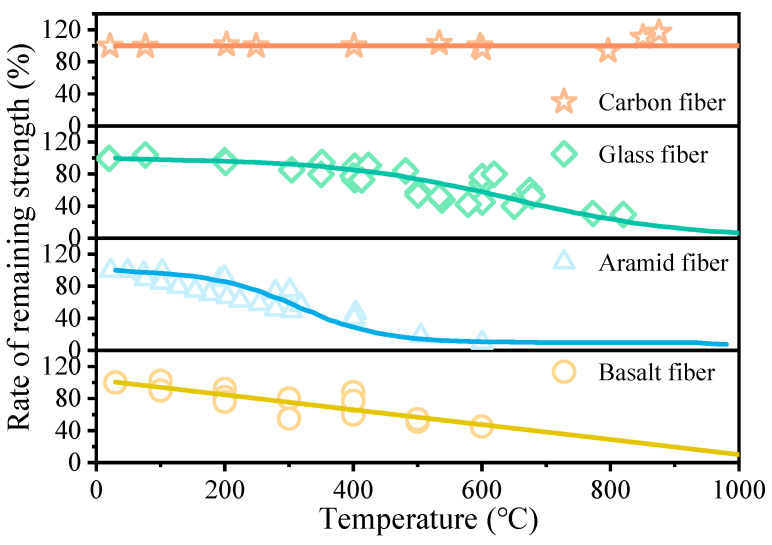
Variation in tensile strength of various fibers with temperature [28,29,30,31,32,33,34,35].

The excellent room-temperature performance of FRP composites is well-established in the literature [21,22,23]. Current research efforts are heavily focused on characterizing their mechanical properties at high temperatures. The thermo-mechanical stability of the reinforcing fibers is critically dependent on the environment. Carbon fibers exhibit superior performance, retaining good mechanical properties at elevated temperatures in an anaerobic environment. However, in the presence of oxygen, carbon fibers undergo oxidation at approximately 400 °C [24]. Prior investigations indicate that carbon fibers can maintain their strength even at temperatures between 1500 °C and 2000 °C when oxygen is excluded [24,25]. Glass fibers, while not susceptible to oxidation, begin to soften at temperatures ranging from 800 °C to 1000 °C [26]. Figure 2 illustrates the influence of elevated temperatures on the tensile strength of various fiber types under anaerobic or inert conditions. Aramid fiber displays the most severe degradation, experiencing a strength reduction of over 50% at 400 °C, whereas carbon fiber shows negligible change. The high-temperature performance of glass fiber is positioned between that of aramid and carbon fibers. Basalt fiber, an emerging reinforcement material, has attracted significant attention due to its favorable properties, including a purported wide operating temperature range of 260 °C to 400 °C. Its tensile strength exhibits a clear trend of linear degradation with increasing temperature [27,28,29,30]. Additionally, natural fibers such as jute, sisal, and hemp are being explored in the context of ‘green’ composites. While these natural fibers offer environmental benefits and low density, their inherent susceptibility to moisture and relatively low thermal decomposition temperatures limit their current application in load-bearing FRP-RC structures. Therefore, this review focuses primarily on the most widely utilized synthetic fibers—carbon and glass—which constitute the mainstream materials for FRP-reinforced concrete structures.

### 2.2. Mechanical Properties of Organic Matrix at Elevated Temperature

The glass transition temperature T_g_ is a critical parameter for quantifying the thermal stability of polymer resins. When the ambient temperature approaches or exceeds T_g_, the resin transitions from a rigid, glassy state to a viscous, rubbery state, leading to a precipitous decline in both strength and elastic modulus [36]. In civil engineering applications, such degradation of the matrix properties fundamentally implies the failure of the force transfer mechanism between the FRP composite and the concrete substrate [24,33,36,37,38,39,40].

**Figure 3:**
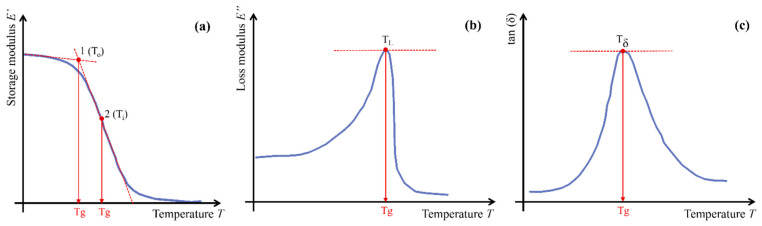
(**a**–**c**) Temperature dependence of viscoelastic parameters and common methods for T_g_ determination via DMA.

The T_g_ of resins is typically characterized using thermal analysis techniques, specifically Differential Scanning Calorimetry (DSC) and Dynamic Mechanical Analysis (DMA). On a DSC curve, T_g_ manifests as a step-type transition associated with changes in specific heat capacity. DMA is employed to evaluate the viscoelastic properties of the resin, focusing on the evolution of the storage modulus *E*′, loss modulus *E*″, and loss factor (tan δ) with temperature [41]. As illustrated in Figure 3, four common methods are used to define T_g_ based on DMA curves: (1) Inflection Point Method (Figure 3a(1)), defined by the inflection point of the *E*′ curve, as per ISO 6721-11 [42]; (2) Tangent Line Method (Figure 3a(2)), defined by the intersection of tangents drawn to the glassy and rubbery regions of the *E*′ curve, as recommended by ASTM E1640-18, DIN EN 61006, and DIN 65583 [35,36,37,42]; (3) Loss Modulus Peak Method (Figure 3b), defined by the temperature corresponding to the peak value of the *E*″ curve [43]; (4) Loss Factor Peak Method (Figure 3c), defined by the temperature at the maximum value of tan δ [43,44,45,46].

Various thermosetting resins can serve as matrices in structural FRP production. Furan, silicone, and cyanate ester resins exhibit exceptional thermal stability and high T_g_ values (exceeding 300 °C in some cases); furan resins, in particular, offer high char yield and low smoke toxicity during fire exposure. However, due to their brittleness, processing complexities, and prohibitive costs, these specialty resins are not mainstream materials in civil engineering and are largely restricted to aerospace or extreme high-temperature industrial components [47,48,49]. In contrast, epoxy, polyester, and vinyl ester resins are widely utilized in civil engineering. Polyester resins are often selected for GFRP applications due to their cost-effectiveness, though they suffer from high curing shrinkage and poor thermal resistance. Vinyl ester resins offer a balanced combination of corrosion resistance and processability. Nevertheless, epoxy resin remains the predominant matrix owing to its high mechanical strength, superior bonding capacity, and low curing shrinkage. Consequently, the fire performance of most commercial FRP systems depends primarily on the thermal properties of the epoxy matrix, which is the focus of this review.

**Figure 4:**
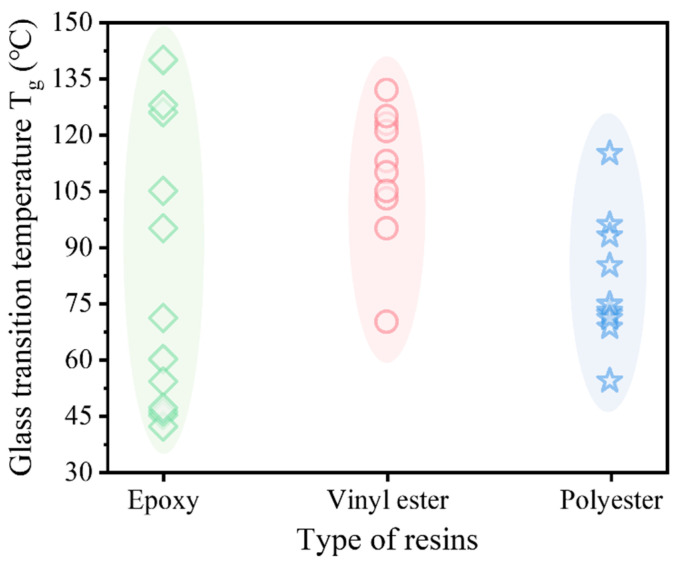
Comparison of T_g_ values of resins obtained using different evaluation methods [36,39,51,52,53,54,55,56,57].

Figure 4 summarizes the T_g_ values of three common resins measured via the inflection point method. Significant fluctuations are observed in all three types, with epoxy resin achieving the highest T_g_ (near 140 °C). Due to the diversity of base materials and additives, T_g_ varies significantly across different formulations; for epoxy resins, the discrepancy between maximum and minimum values reaches nearly 100 °C. Even within a single formulation, T_g_ is sensitive to curing conditions, testing methods, and thermal history. Michels et al. [39] demonstrated that curing procedures and specimen age have the most significant impact on T_g_, while thermal history has a minimal effect (<10%). High-temperature accelerated curing can increase T_g_ by approximately 20%. As the curing age extends from 3 to 28 days, T_g_ rises from 53.1 °C to 67.9 °C. This improvement is attributed to the continuous cross-linking of residual reactive groups, which increases the cross-link density and restricts polymer segmental mobility. Ke et al. [36] found that T_g_ values obtained from different evaluation methods can differ by over 10 °C, with the tangent method typically yielding the highest results and the loss factor method the lowest. Tensile tests further confirm that epoxy resin properties deteriorate significantly even at moderate temperatures. When the temperature rises from 25 °C to 55 °C, tensile strength decreases by 26–72% and the modulus by 21–94%. Yu et al. [33] reported that degradation accelerates significantly above 60 °C, with a 73.08% loss in strength at 100 °C. Plecnik et al. [50] noted that materials lose almost all load-bearing capacity at temperatures approximately 100 °C above T_g_.

Current research consistently indicates that the T_g_ of epoxy resins used in civil engineering is typically below 100 °C, which is substantially lower than temperatures encountered during fire events. Since significant performance loss occurs even below T_g_, developing advanced bonding technologies that increase T_g_ while maintaining cost-efficiency remains a vital direction for future research.

### 2.3. Mechanical Properties of FRP Composite Structures

As mentioned above, FRP is mainly used in civil engineering in two forms, strengthened or reinforced materials, regardless of which elevated temperature will cause a significant reduction in its performance. Up to now, many experiments have been conducted to investigate the effect of elevated temperature on the mechanical properties of FRP composited structures. This part aims at reviewing some of the experimental and analytical investigations reported in recent years.

#### 2.3.1. FRP-Based External Strengthening Materials

**Figure 5:**
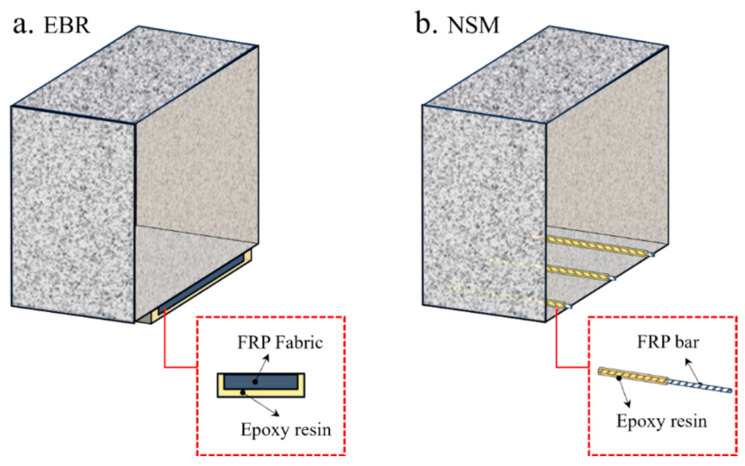
Common FRP strengthening techniques: (**a**) EBR; and (**b**) NSM.

Maintenance and strengthening of existing building structures have increasingly become major engineering priorities, particularly in developed countries [58]. Compared with traditional strengthening materials, FRPs offer several notable advantages: (1) excellent resistance to environmental degradation, (2) high specific stiffness and strength, and (3) ease of installation [59]. The commonly adopted FRP strengthening techniques are illustrated in Figure 5. The external bonding reinforcement (EBR) technique involves directly bonding FRP sheets or plates to the surface of structural members using epoxy adhesive, as shown in Figure 5a. Although widely used, the EBR method is prone to premature debonding at relatively low FRP strain levels, which prevents full utilization of the material’s tensile capacity. This drawback has motivated the development and increasing use of the near-surface mounted (NSM) technique. In the NSM method, FRP bars or strips are embedded in pre-cut grooves on the surface of the member and bonded using epoxy resin or grout, as illustrated in Figure 5b [60]. This technique offers superior bond performance and a higher load-carrying capacity compared with the EBR method, as the FRP is embedded in grooves and therefore less prone to premature debonding. Moreover, the NSM method enables the installation of a larger amount of FRP material, thereby improving the overall strengthening efficiency. The epoxy adhesive not only ensures effective bonding between the FRP and the substrate but also provides protection against environmental degradation, contributing to enhanced long-term durability. Appropriate groove dimensions and alignment are essential to ensure efficient stress transfer and to prevent damage to the existing structure during installation. Previous studies have demonstrated that NSM-strengthened members exhibit improved flexural and shear behavior, accompanied by minimal interface slip.

At room temperature, FRP systems can substantially enhance the load-bearing capacity of structural members and extend their service life, enabling the adaptive reuse of buildings and the restoration of deteriorated structures to their original design performance. However, the additional capacity provided by FRP deteriorates rapidly under elevated-temperature conditions. This degradation is primarily attributed to the loss of anchorage in the epoxy adhesive, which prevents the FRP composite from effectively contributing to load resistance. Although this shortcoming has been extensively documented in the literature, a reliable solution to the poor high-temperature performance of FRP systems has yet to be developed. At present, the common engineering practice is to apply a fire protection layer of sufficient thickness over the FRP. Research on the strengthening of steel reinforced concrete (RC) structures—such as beams, columns, and slabs—using FRP at room temperature is well established, and corresponding design codes and guidelines have been issued in many countries [61].

The limited thermal resistance of FRP composites remains a major obstacle to their widespread application in structural engineering. Numerous studies have investigated the behavior of FRP-strengthened concrete elements exposed to elevated temperatures. Table 2 summarizes the available research on the mechanical performance of FRP–concrete composite systems under high-temperature or fire conditions, including both experimental and numerical investigations.

Without protection, EBR FRP systems exhibit rapid debonding as temperature increases. However, specimens do not necessarily fail immediately after debonding due to two mechanisms: (1) residual anchorage at the FRP termination, which activates a cable-like action, and (2) the ability of the original RC section to sustain loads exceeding the applied level when the external load is relatively low. Previous studies reported that this cable mechanism can extend the fire resistance of FRP-strengthened members by up to 37 min. In contrast, specimens subjected to higher load levels fail abruptly once the FRP detaches from the concrete substrate [62]. For FRP-strengthened beams protected with fire-resistant coatings, a shift in failure mode may occur at elevated temperatures—from flexural failure to shear-dominated failure [69]. When adequately insulated, FRP-strengthened beams can withstand the ASTM E119 standard fire curve or a typical design fire scenario for several hours [62,68,69]. Additionally, boundary restraints influence elevated-temperature performance of the beam. Axial restraint, which limits the thermal expansion of the beam, induces arching action and generates a counteracting moment, resulting in reduced midspan deflections [68].

**Figure 6:**
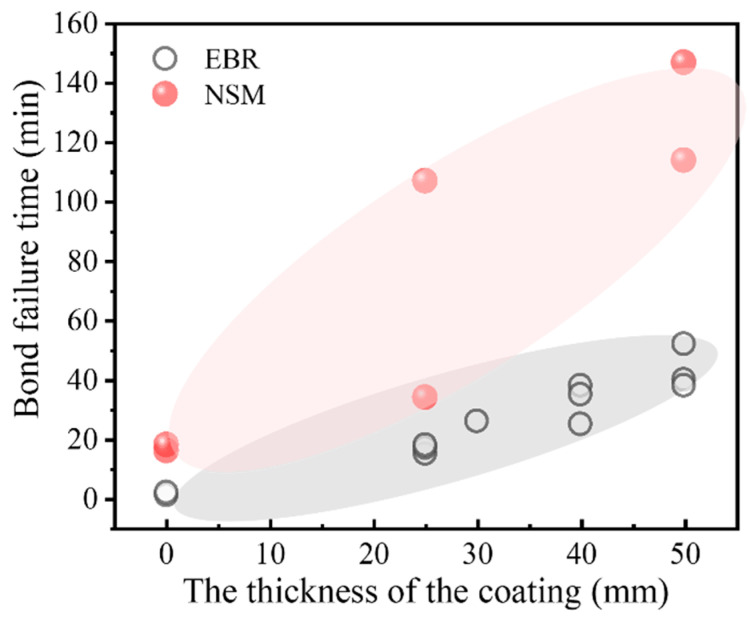
Comparison of FRP debonding time in EBR- and NSM-strengthened RC beams under elevated-temperature exposure [62,69,70,71].

The EBR technique has become the most widely applied method for strengthening RC beams and columns. However, because EBR leaves the FRP externally exposed, it is more vulnerable to heat compared with the NSM method. Consequently, NSM strengthening is considered a viable alternative for high-temperature applications. To examine the thermal performance of different strengthening configurations, several researchers have conducted experimental programs. Yu et al. [66] tested four T-section RC beams strengthened with NSM method. Their fire tests demonstrated that NSM-strengthened beams could sustain service loads for up to 3 h under standard fire exposure, even without external insulation. When FRP-strengthened beams are provided with sufficient fire protection, the strengthening technique (EBR versus NSM) has minimal influence on the overall fire resistance [66]. Axial restraint was shown to extend the fire resistance of NSM-strengthened beams, whereas higher load ratios had an adverse effect. Firmo et al. [62,69,70,71] carried out a series of fire resistance tests to examine the influence of strengthening configuration (EBR or NSM), fire protection scheme, and adhesive type (epoxy resin or grout) on the performance of FRP-strengthened RC beams. As shown in Figure 6, FRP debonding in the NSM-strengthened specimens occurred later than in the EBR-strengthened specimens.

For the EBR system, debonding initiated at much lower temperatures within the anchorage zone, typically only slightly above the adhesive T_g_. In contrast, NSM systems exhibited substantially higher critical temperatures at the anchorage interface. When the FRP does not rupture at high temperature and the anchorage zone remains relatively cool, partially deboned FRP can engage in a cable mechanism. With this additional load-transfer mechanism, the fire resistance of FRP-strengthened beams can reach approximately 2 h. In addition, Palmieri et al. [72,73] and Zhu et al. [74] investigated the fire performance of NSM strengthening systems. Their findings consistently show that the NSM technique exhibits superior fire resistance compared with the EBR method. This improvement is primarily attributed to the partial embedment of the FRP materials within the concrete, which provides inherent thermal protection to both the FRP and the adhesive under elevated-temperature exposure. The effectiveness of FRP strengthening at high temperatures is closely tied to the anchorage behavior between the FRP and the concrete substrate. Adriana [75] conducted single-lap shear tests on concrete prisms strengthened with CFRP strips inserted into pre-cut grooves and bonded with an epoxy adhesive. Specimens were heated to temperatures up to 270 °C and subsequently loaded to failure. The results demonstrated that both bond strength and bond stiffness decrease significantly as temperature increases. The strain distribution along the bonded length transitioned from a markedly nonlinear profile at temperatures below 50 °C to an almost linear distribution at higher temperatures. Correspondingly, the failure mode shifted from CFRP tensile rupture at 20 °C and 50 °C to adhesive failure at the CFRP–adhesive interface at elevated temperatures. Firmo et al. [76] performed double-lap shear tests on concrete blocks externally bonded with CFRP strips using epoxy adhesive. Two loading protocols were employed: (1) specimens were heated to the target temperature and then loaded to failure; (2) specimens were pre-loaded to 25%, 50%, or 75% of their ambient-temperature capacity before being heated to failure. The results show that mechanical anchorage enhances bond strength across all tested temperatures; however, specimens with mechanical anchorage exhibited similar rates of strength degradation with increasing temperature compared with those without such detailing. The temperature-induced changes in strain distributions mirrored the trends observed in Adriana’s study. Azevedo et al. [71] examined the fire behavior of RC slabs strengthened with CFRP strips using three different techniques: EBR, NSM, and continuous reinforcement embedded at the ends (CREatE). The results clearly indicate that both NSM and CREatE systems, with or without protective fire insulation, outperform the EBR technique in terms of fire resistance, which is consistent with the aforementioned findings.

#### 2.3.2. FRP Reinforcing Bars

The combination of steel bar and concrete shows many excellent properties, but the corrosion of steel bar remains an unresolved issue, which is also a key factor determining the durability of RC structures. FRP composite materials not only possess good performance in strengthening and repairing RC structures, but also exhibit great potential for improving the durability of RC. Due to the different composition between FRP composite and steel, the performance of FRP at elevated temperature is also different from steel. Furthermore, concrete members reinforced with FRP and steel show different fire resistance ability. For example, a fully loaded, simply supported steel-reinforced concrete beam, subjected to a standard fire under full-service loads, will be approaching its collapse load when the flexural steel reaches temperatures in the range of 540–600 °C [77]. This is due to the fact that both concrete and reinforcement undergo significant performance degradation in this temperature range, such as strength, modulus of elasticity, etc. [78]. Unlike steel, epoxy resin is an indispensable part of the manufacturing process of FRP composites, so FRP composites are more sensitive to temperature which causes the deterioration of mechanical and bonding properties. Numerous studies [79,80,81] have shown that CFRP, GFRP, and AFRP suffer deterioration of tensile strength and bond properties at temperatures between 100 °C and 350 °C, and the degree of deterioration varies with the raw material of the FRP and surface treatment methods. To date, many studies have focused on the mechanical properties of FRP-reinforced concrete (FRP-RC) elements at room temperature [3,14,82,83], and relatively limited studies on the fire resistance of FRP-RC elements.

Limited test programs about the fire resistance of FRP-RC structures have been reported. Zhao et al. [84] investigated the fatigue behavior of concrete beams reinforced with FRP bars after exposure to elevated temperatures. A total of 13 concrete beams (150 mm × 200 mm × 1800 mm) reinforced with CFRP/GFRP bars were tested. The beams were heated to the target temperatures and sustained for 1, 2, or 3 h. After cooling to room temperature, fatigue tests were conducted. The results indicated that at 400 °C, the fatigue life of CFRP-reinforced concrete beams was only slightly affected; notably, it remained almost three times longer than that of their GFRP-reinforced counterparts. However, the load-bearing capacity of both CFRP- and GFRP-reinforced beams was nearly lost at 600 °C. Rajai et al. [85] casted 50 beams of 100 mm × 150 mm × 1100 mm in size and carried out four-point bending tests before and after exposure to elevated temperatures to investigate the feasibility and effectiveness of CFRP strips as a substitute for stirrups. Prior to the four-point bending test, the specimen was heated to 150–500 °C and kept at a constant temperature for 2 h, then cooled in the furnace to room temperature. Since no debonding occurs, the CFRP strips inside the beam can provide a higher shear load capacity than using CFRP strips as externally bonded strips, both at room temperature and elevated temperature. Tian et al. [86] conducted a study on the fire resistance of hybrid-reinforced concrete beams (steel and GFRP bars) and the undamaged beams were subjected to four-point bending test after 21 days of fire resistance test. After exposure to elevated temperatures and cooling to room temperature, steel bars partially recovered their mechanical properties due to metallic grain realignment, whereas GFRP bars exhibited no recovery owing to irreversible resin decomposition and fiber-matrix debonding. Therefore, the bearing capacity of the specimen gradually decreased as the reinforcement ratio of GFRP bars increased, and the GFRP bars were ruptured as the ultimate load was reached. Sakashita et al. [87] conducted fire tests on 11 concrete beams reinforced with FRP bars to study the effect of fiber types (carbon, glass and aramid) and fabrication method (spiral, braided and straight) on the fire resistance of the beams. The test results indicated that the fire resistance of FRP -RC beams decreased in order of CFRP, GFRP and AFRP. In addition, beams with spiral or straight fiber bars showed superior fire resistance because their direct load paths are less matrix-dependent. In contrast, braided structures rely on interlacing pressures that fail rapidly during thermal softening, leading to fiber slippage [87].

The anchorage performance between steel bars and concrete is known to strongly influence the mechanical behavior of reinforced concrete members, and the same principle applies to FRP bars. The bond mechanism between FRP bars and concrete relies primarily on mechanical interlocking and surface friction. Unlike steel bars, where surface deformations are integral to the high-stiffness metallic core, the interlocking mechanism of FRP bars is governed by the polymer resin at the interface. When an FRP-reinforced concrete member is exposed to fire, the temperature at the bar surface rapidly exceeds the T_g_ of the epoxy resin. This causes the resin to soften and lose its ability to transfer shear stresses between the bar and the concrete, leading to a much sharper reduction in bond strength compared to steel reinforcements. Consequently, the effectiveness of mechanical interlocking and friction depends on the properties of the surface epoxy resin and the surface treatments applied to the bars (e.g., sand coating, helical wrapping, molded deformations). When an FRP-reinforced concrete member is exposed to fire, the temperature at the FRP bar rapidly exceeds the T_g_ of the epoxy resin, leading to a sharp reduction in bond strength between the bar and the surrounding concrete. Therefore, a thorough understanding of the bond behavior between FRP bars and concrete under elevated temperatures is essential for assessing the fire resistance of FRP-reinforced concrete structures. Rami et al. [79] investigated the bond behavior between concrete and different types of FRP bars (CFRP, GFRP, and AFRP) after exposure to temperatures of 125, 250, 325, and 375 °C for 3 h. The results showed that the bond strength between steel bars and concrete at elevated temperatures remained significantly higher than that of FRP bars. After exposure to 250 °C, steel bars retained the highest bond strength at 9.06 MPa, followed by CFRP, BFRP, and GFRP at 4.7 MPa, 1.03 MPa, and 1.00 MPa, respectively. Among the FRP bars, CFRP exhibited the highest bond strength due to its superior surface characteristics (e.g., sand-coated finish). At 325 °C, the bond between FRP bars and concrete deteriorated substantially, with residual strengths dropping to approximately 20% of their original values. Surface treatment plays a critical role in the bond performance of FRP bars. At room temperature, ribbed FRP bars generally achieve better bond behavior than sand-coated bars. However, as temperature increases from 20 °C to 80 °C, sand-coated FRP bars retain more than 90% of their original bond strength (or show a slight increase), whereas ribbed bars experience a pronounced reduction [88,89]. This is because the epoxy resin softens and loses strength with increasing temperature, while the sand coating continues to provide surface friction, thereby preserving a portion of the bond capacity. Table 3 provides a summary of studies investigating the effect of elevated temperatures on the bond performance between FRP bars and concrete.

### 2.4. Summary

As reviewed above, numerous experimental studies have investigated the fire resistance of FRP-reinforced concrete members. However, variations in specimen size, type of fiber used in FRPs, manufacturing processes, and test configurations limit the direct comparability of their results. Epoxy resin, an essential component in both FRP composites and FRP-concrete bonded systems, exhibits substantial mechanical degradation at relatively moderate temperatures (100–200 °C). This deterioration critically reduces the high-temperature performance of FRP composites and undermines the integrity of FRP–concrete interfaces. Implementing appropriate fire-protection measures, particularly in the FRP anchorage zones, can delay temperature rise and promote the development of the FRP cable mechanism, thereby significantly enhancing the fire endurance of the FRP-concrete system. Nonetheless, at elevated temperatures, the bond performance between FRP bars and concrete remains distinctly inferior to that of ribbed steel bars. Furthermore, while steel bars exposed to fire can partially recover their mechanical properties after cooling, FRP bars lack this recovery capacity due to the irreversible thermal degradation of their polymer matrix. Surface treatment plays an important role in the bonding behavior of FRP bars, with sand-coated bars demonstrating comparatively superior bond retention at elevated temperatures. However, despite these enhancements, the anchorage performance of FRP bars deteriorates sharply and becomes almost entirely ineffective once the temperature approaches approximately 300 °C.

## 3. Strategies for Enhancing Fire Resistance of FRP Structures

As discussed in Section 2.1, carbon fibers retain stable mechanical properties at elevated temperatures, and glass fibers maintain satisfactory performance up to approximately 400 °C. In contrast, epoxy resin is highly temperature-sensitive, exhibiting a rapid decline in mechanical properties as temperature increases. Because epoxy resin functions as the primary binder in the EBR strengthening technique—facilitating stress transfer between the FRP and the concrete substrate—the thermal performance of FRP composites is ultimately governed by the thermal stability of the epoxy adhesive.

To overcome these thermal limitations, both organic and inorganic binders have been explored as alternative stress-transfer media. Organic binders, dominated by epoxy resins, remain the mainstream choice due to their superior tensile strength and interfacial adhesion. Their reinforcement mechanism relies on the chemical cross-linking of polymer chains to form a dense network, providing robust micro-mechanical interlocking and chemical bonding with the substrate. To enhance their heat resistance, various modification strategies for the resin matrix, curing agents, and fillers have been developed. Matrix modification typically involves increasing cross-linking density or blending with thermally stable polymers (e.g., phenolic or silicone resins). Additionally, incorporating heat-resistant fillers—such as silica, carbon nanotubes, or phosphates—can improve thermal stability, provided they are uniformly dispersed to avoid impairing mechanical integrity [85]. However, the primary drawback of organic binders is their low T_g_, beyond which bond integrity fails precipitously.

In contrast, inorganic binders (e.g., cementitious mortars or geopolymer pastes) offer a fundamentally different mechanism based on a stable mineral crystalline matrix, such as calcium silicate hydrate (C-S-H). Unlike the chemical bonding in epoxies, inorganic binders achieve adhesion through mechanical interlocking and physical suction within the porous concrete [92]. The primary advantages of inorganic binders include exceptional fire resistance (stable above 600 °C), lack of toxic fumes, and excellent thermal compatibility with concrete. Nevertheless, their limitations are significant: they exhibit lower interfacial shear strength than epoxies, often necessitating larger bonding areas or specialized FRP surface treatments to prevent brittle debonding.

### 3.1. Comparative Analysis of Organic and Inorganic Binders

To date, numerous heat-resistant resin adhesives have been developed; however, most are unsuitable for civil engineering applications due to their high cost, complex manufacturing processes, or both. For example, Performance Polymer Solutions Inc. has developed an organic–inorganic hybrid polyimide resin with a T_g_ as high as 489 °C [93], yet such materials are impractical for large-scale structural applications. Due to the mechanical properties of epoxy, resins deteriorate significantly at elevated temperatures, the use of FRP composites and epoxy-based bonding systems is inherently restricted to service conditions below the T_g_ of the adhesive. Additionally, epoxy resins are combustible and can contribute to flame spread and toxic smoke generation during fire exposure. These drawbacks motivate the search for alternative bonding systems with superior thermal stability. To address the limitations of epoxy-based systems at high temperatures, extensive research has focused on enhancing the heat resistance of FRP-resin adhesive systems. One promising approach is to replace epoxy resin with inorganic adhesives in FRP strengthening applications, as these materials offer superior thermal and fire resistance. In the following section, the bonding performance of inorganic adhesives is compared with that of epoxy resin adhesives under room- and high-temperature conditions.

#### 3.1.1. Bonding Strength with Beam

Table 4 summarizes several studies comparing the strengthening performance of inorganic and organic adhesives. Overall, inorganic adhesives provide bonding performance at room temperature comparable to that of organic (epoxy-based) adhesives [75,94,95,96,97]. However, beams strengthened with organic adhesives generally exhibit greater deformation capacity due to the intrinsically higher ductility of polymer-based systems [96]. Regardless of the adhesive type, the ductility coefficients of FRP-strengthened beams decrease markedly relative to unstrengthened reference beams [95]. The smaller ultimate deformations observed in [94] are attributed to the use of short plain concrete beams.

**Figure 7:**
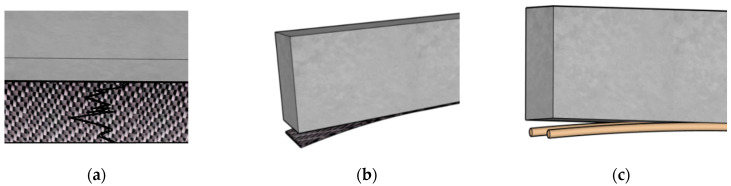
Failure modes of FRP strengthening systems: (**a**) FRP rupture in EBR; (**b**) FRP debonding in EBR; and (**c**) failure mode of the NSM-FRP strengthening system.

In EBR systems, the predominant failure mode differs significantly between adhesive types. Beams strengthened with inorganic adhesives tend to fail by tensile rupture of the FRP, whereas those bonded with organic adhesives typically fail by debonding at the FRP-concrete interface. These failure morphologies are illustrated in Figure 7a,b, and the corresponding quantitative data are summarized in Table 4. However, as the number of FRP layers increases, the bond capacity of inorganic adhesives can become insufficient to sustain FRP rupture, resulting in a shift toward interface debonding [95]. The comparative studies by Deng, Toutanji, Zhao and colleagues [96,97] found that beams bonded with geopolymer adhesives exhibit deformation capacities similar to those bonded with epoxy resin, though both remain lower than those of unstrengthened beams. Both adhesive types provided comparable strengthening effects. Kurtz et al. [95] further demonstrated that adhesive type directly affects failure mode: epoxy-bonded beams failed by CFRP delamination, whereas beams bonded with alkali-activated cementitious materials failed through CFRP tensile rupture. Similar conclusions were reported by Chen and Wang et al. [75,94], reinforcing that strengthening effectiveness is comparable across adhesive types, but inorganic adhesives lead to lower ultimate deflection because of their limited deformability.

In NSM systems, failure modes are more varied due to differences in FRP composite types [98,99,100]. Compared with EBR-FRP beams, NSM-FRP strengthened beams more frequently exhibit debonding at the interface between the groove and the inorganic adhesive, as shown in (Figure 7c). Experimental findings in [99] further indicate that RC beams repaired and strengthened with NSM CFRP strips using a high-strength self-compacting cementitious adhesive—composed of cement, sand, and admixtures—achieved significant improvements in both ultimate flexural capacity and ductility compared to the reference beams. This demonstrates that well-designed inorganic adhesives can match—and in some cases exceed—the performance of organic adhesives in NSM applications. Taken together, these studies show that inorganic adhesives offer bonding performance comparable to organic adhesives at room temperature and exhibit favorable failure behaviors in both EBR and NSM systems. However, their inherently lower deformation capacity leads to reduced ultimate deflection in strengthened beams. Nonetheless, advances in modified inorganic adhesives show strong potential for enhancing both strength and ductility in FRP strengthening applications.

**Table 4:** Comparative performance of inorganic and organic adhesives in FRP strengthening.

Ref	Fiber Layer	Adhesive Type	F*_y_* (kN)	F*_u_* (kN)	Δ*_y_* (mm)	Δ*_u_* (mm)	Δ*_u_*/Δ*_y_*	Failure Mode
[96]	2	Epoxy resin	18.9	27.8	7.3	15.6	2.1	FD
	3		19.9	27.9	7.6	15.9	2.1	
	4		22.3	32.9	8.3	16.9	2.0	
	2	Geopolymer	18.5	22.6	6.5	10.7	1.6	FR
	3		22.9	27.8	7.7	14.5	1.9	
	4		25.3	32.8	8.5	15.6	1.8	
[100]	3	Cement	40.7	52.9	7.1	12.4	1.8	FR
	4		45.5	55.4	7.0	13.1	1.9	FR
	5		50.1	62.3	6.8	13.2	2.0	FD
	6		52.3	63.2	7.2	12.1	1.7	FD
[95]	0	Cement	57.8	74.5	11.0	94.0	8.55	Flexural
	2		73.4	80.5	13.0	20.1	1.6	FR
	3		75.6	91.9	12.9	23.3	1.8	FR
	5		84.5	110.1	14.0	24.1	1.7	FR
[75]	1	Epoxy resin	185	220	7.0	14.0	2	FR
	1	Geopolymer	200	230	7.3	11.5	1.6	FR
	2		245	270	8.0	11.2	1.4	FR
[94]	1	Magnesium phosphate cement		34.6		0.508		Flexural
		Magnesium oxychloride cement		34.3		0.57		Flexural
		Geopolymer		21.2		0.155		Flexural
		Polymer-modified mortar		33.5		0.404		Flexural
		Epoxy resin		53.3		1.018		Shear
[98]	NSM	Epoxy resin	36.8	53.3				Rod pull-out
		Mortar	35.3	43.9				Debonding at adhesive-concrete interface
[101]	NSM	Epoxy resin		150.7		34.8		Debonding of concrete cover
[99]		Self-compacting cement		134.3		106.4		FR
[100]	NSM	Cement	93.9	114.5		35.7		FD
			127.9	140.9		22.9		FD

Note: F*_y_* and F*_u_* represent the yield and ultimate loads, respectively; Δ*_y_* and Δ*_u_* denote the corresponding displacements at the yield and ultimate stages; Δ*_u_*/Δ*_y_* is the displacement ductility ratio. FD denotes fiber debonding; FR denotes fiber rupture.

#### 3.1.2. Effect of High Temperature on Bonding Properties

To address the limitations of epoxy-based bonding systems at elevated temperatures, the use of inorganic adhesives for bonding FRP composites has been proposed as an effective alternative to enhance the thermal resistance of FRP strengthening systems. Because the bond behavior at the FRP–concrete interface governs the overall strengthening efficiency under high-temperature conditions, special attention must be devoted to understanding the performance of inorganic adhesives at elevated temperatures. However, research on the high-temperature behavior of CFRP-strengthened concrete members using inorganic adhesives remains limited.

Table 5 summarizes existing studies on the influence of elevated temperature on the bonding properties of inorganic adhesives. Due to variations in FRP type, anchorage length, testing procedures, and other experimental parameters, the comparability among test results is limited. Nevertheless, in general, the reduction in bond strength with increasing temperature is less pronounced for inorganic adhesives than for organic adhesives in NSM-FRP strengthening systems. Studies reported somewhat contradictory results, likely attributable to differences in load levels applied at the FRP ends [102,103].

**Figure 8:**
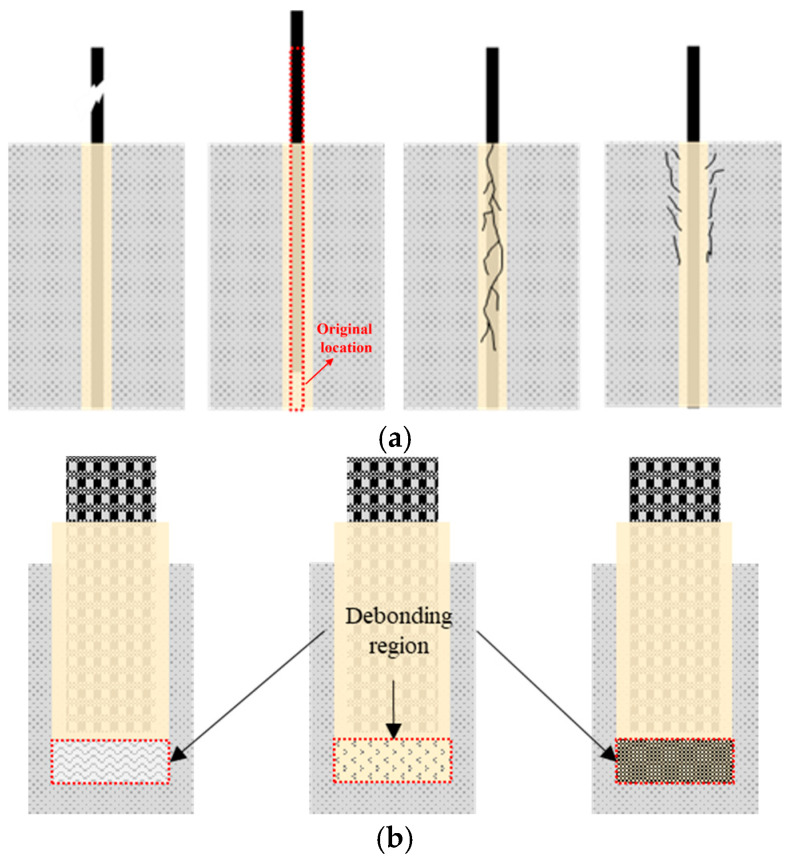
Bonding failure modes of FRP-concrete under elevated temperature: (**a**) NSM; (**b**) EBR.

Experimental findings in [101] further indicate that applying an innovative high-strength self-compacting cementitious (HS-SCC) coating can substantially mitigate temperature-induced bond degradation in inorganic adhesives, enabling improved bond retention after exposure to elevated temperatures. Surface treatment of CFRP composites is also an effective method for enhancing high-temperature bonding performance. For example, FRP coated with a layer of coarse sand (particle size 0.6–0.85 mm) exhibited better bond performance with inorganic adhesive at elevated temperatures than FRP coated with fine sand (maximum particle size 0.2 mm) [104]. In EBR-FRP strengthening systems, inorganic adhesives continue to demonstrate superior high-temperature bond performance compared with organic adhesives. The corresponding failure modes under elevated temperatures are shown in Figure 8. In NSM-FRP systems, elevated-temperature failure is primarily initiated by damage at the interface between the FRP composite and the inorganic adhesive, which differs from the failure mode observed at room temperature [101,102,103]. Since the surface of FRP composites is typically coated with epoxy resin, the rapid thermal degradation of epoxy at elevated temperatures leads to significant weakening of the epoxy–inorganic adhesive interface, ultimately triggering system failure. In EBR-FRP systems, elevated-temperature failure generally occurs through interlaminar slip within the FRP composite, whereas specimens tested at ambient temperature predominantly show minor mortar detachment or FRP rupture [105].

Based on the research of predecessors, inorganic adhesives can improve bonding performance of FRP reinforcement systems at the high temperature. However, inorganic adhesives are prone to cracking under elevated temperatures, potentially causing premature failure of FRP strengthening systems. None of the current studies consider the thermal deformation mismatch between the inorganic adhesive and concrete, which will lead to the damage of the interface between the inorganic adhesive and concrete.

**Table 5:** Summary of studies on the elevated-temperature bond performance of inorganic adhesives used in FRP–concrete strengthening systems.

Ref	Dimension of Specimen (mm)	Concrete			Adhesive			FRP						*F* (kN)	*T* (°C)	Exposure Duration (min)
		*f_cc_* (MPa)	*f_tc_* (MPa)	Groove *w* × *h* (mm)	*f_ca_* (MPa)	*f_ta_* (MPa)	Type	Type	*f_fu_* (MPa)	*E_f_* (GPa)	Δ (%)	Cross-Section *w* × *t* (mm)	Bonding Length (mm)			
[102]	60 × 60 × 100	C25/C30		4 × 11	N/A	N/A	Inorganic	CFRP plate	2800	165		10 × 1.2	70/90	0.40	530	20.22
														0.84	632.6	23.3
					N/A	81.4	Organic							0.40	837.8	
														0.84	543.7	
[103]	75 × 75 × 250	41	3.84	4 × 18	88	6.2	Inorganic	CFRP textile	1500	135		14 × 1.5	50	4.57	240	N/A
													100	5.62	250	
					60	30	Organic						50	5.67	140	
[101]	75 × 75 × 250	32	3.6	5 × 30	113	14.3	Inorganic	CFRP strip	3697	210	1.74	20 × 1.52	180	34.53	21	N/A
														21.24	400	
														6.53	600	
														3.14	700	
														2.82	800	
[104]	100 × 100 × 200			10 × 20	78.59		Inorganic	CFRP	1909	171		10 × 1.4	125	6	383.7	
														6	455.5	
[105]	40 × 40 × 160	49.7	N/A	EBR	N/A	N/A	Inorganic Organic	CFRP sheet	3085	N/A	N/A	40 (wide)	30	3.61	20	N/A
														1.79	100	
														1.73	200	
														1.74	300	
														3.94	20	
														1.51	50	
														0.76	100	
														0.11	150	
[75]	100 × 100 × 100	30.95	N/A	EBR	35	N/A	N/A	CFRP	4125	244	1.71	70 × 0.167	100	13.7	RT	N/A
														9.87	105	
														8.89	200	
														7.49	300	
														5.53	400	
														2.87	500	
[106]	160 × 160 × 1500	33.7	N/A	EBR	80.88	3.24	N/A	CFRP	4125	244	1.71	70 × 0.167	325	11.61	100	N/A
													325	10.55	200	
													325	13.98	300	
													325	10.67	400	
													300	12.59	500	

#### 3.1.3. Thermal Performance of FRP Strengthening Beam

The effectiveness of FRP strengthening technique depends on the bond behavior between the concrete and FRP composites. Direct pull-out tests, including single- or double-lap shear tests, have been carried out to assess the bond behavior between the concrete and FRP. Nevertheless, bending tests of FRP-strengthening beams offer a more comprehensive understanding of the actual bond behavior between concrete and FRP. Some researchers conducted bending tests at elevated temperatures to study the fire endurance of FRP-strengthening beams. Table 6 summarizes several studies about the effect of elevated temperatures on the performance of FRP strengthening beams with inorganic adhesive.

Elevated temperature tests on FRP-strengthening beams without fire protection were conducted by Hashemi et al. [107]. Various adhesives, including epoxy resin and cementitious type materials, were used to adhere the FRP fabric or textile to the bottom surface of the beams. The results show that the deflection curve of CFRP reinforced specimens with epoxy resin appears two jumping points during heating: the first point at 66 °C, corresponding to initial intermediate debonding, and the second point at 385 °C, marking complete debonding of FRP. However, the test group using inorganic adhesive did not show this phenomenon and exhibited superior heat resistance.

In another similar experiment [108], the beam reinforced by CFRP sheet using epoxy resin as adhesive did not show a sudden increase in deflection curve, attributed to the U-shaped strips that allowed the bottom CFRP sheet to form a cable mechanism. The test results also emphasize the critical role of the fire insulation layer’s integrity in specimen fire resistance. When the fire insulation layer is partially peeled off, even if the geopolymer with better heat resistance is used as adhesive to adhere CFRP, the fire resistance of the CFRP strengthening beam is significantly reduced, even worse than the epoxy resin strengthening beam. In addition, the high temperature resistance of EBR reinforced beams was also experimentally investigated by Zheng, Chen and Zhang et al. [109,110,111]. The inorganic adhesive used in these experiments was alkali-activated slag and MOC (magnesium chloroxylate) cement. Even though the high temperature resistance of inorganic adhesive is much better than epoxy resin, the presence of a fire insulation layer remains crucial to ensure the sustained effectiveness of inorganic adhesives. After fire test, the fire insulation layer on the beam is completely lost, and the FRP composite ruptures at the high moment region. The combined action of load and temperature gradually increases specimen deformation, leading to premature cracking of the fire insulation layer at the high moment region. Without the fire insulation layer to insulate oxygen and heat, the FRP composite undergoes rapid carbonization due to elevated temperature, with the resin essentially volatilizing.

In addition to the EBR strengthening method, the NSM technique is also widely applied in practical engineering. As expected, NSM-strengthened RC beams exhibited significantly superior fire performance compared to EBR-strengthened beams under similar fire exposure, as shown in Figure 6. Firmo et al. [70] conducted fire resistance tests on RC beams strengthened using the NSM method, where CFRP strips were bonded with either a conventional epoxy adhesive (T_g_ = 47 °C) or a mixed grout (T_g_ = 44 °C). The test results show that without insulation, the NSM-CFRP systems failed after 18 min (epoxy) and 17 min (mixed grout), whereas with insulation the failure times increased substantially to 34 min and up to 114 min, depending on the insulation scheme employed. The composition and thermomechanical properties of inorganic adhesives have a marked effect on the elevated-temperature behavior of NSM-FRP systems. In Firmo’s study [70], the inorganic adhesive used had lower tensile strength and lower T_g_ than the organic adhesive, resulting in poorer fire resistance for NSM systems bonded with inorganic adhesive. In contrast, the results reported by another research demonstrated that fiber-reinforced inorganic adhesives maintain better integrity at elevated temperatures, enabling NSM-FRP strengthened beams to achieve enhanced thermal resistance [112]. It is also worth noting that the grooves in the anchorage zone and non-anchorage regions were filled with organic and inorganic adhesives, respectively [112]. This hybrid adhesive strategy provided the strengthened beams with excellent fire resistance. Furthermore, when a sufficiently thick fire-protection layer is applied, NSM-FRP strengthened beams can retain 86–92% of their room-temperature flexural capacity after one hour of high-temperature exposure [73]. Finally, treating the adhesive T_g_ as the critical temperature for the anchorage zone is overly conservative. Based on the findings of [70,73], adopting a critical temperature equal to approximately 2 × T_g_ provides a more realistic and reliable criterion.

**Table 6:** Summary of the application of inorganic adhesives in FRP strengthened beams.

Ref	Dimension of Specimen *l* × *w* × *h* (mm)	Strengthen Technique	FRP Type	Adhesive		Thermal Insulation (mm)	Applied Load/Unstrengthened Ultimate Capacity (%)	Applied Load/Strengthened Ultimate Capacity (%)	Heating Rate (°C/min)	T (°C)	Fire Resistance Period (min)
				*f_ca_* (MPa)	*f_ta_* (MPa)						
[107]	1600 × 120 × 180	EBR	CFRP textile	65	6.8	0	57.8–59.4	41.1–41.9	20		100
[108]	5300 × 250 × 400	EBR	CFRP sheet			10		50	ISO834	80–200	20–60
[110]	3300 × 150 × 300	EBR	CFRP textile	113.3		15		60	ISO834	380–430	150
[70]	1500 × 100 × 120	NSM	CFRP strips		6.6	25	78	37	ISO834	88.5–279	17–114
						50					
[73]	3000 × 200 × 300	NSM	CFRP rod		4.1	20	63–71	37–54	ISO834	163	60 (just heat 1 h)
[112]	3900 × 200 × 450	NSM	CFRP rod	52.4/41.9		0	77.1	50	ISO834		94–126
[113]	2600 × 200 × 300	NSM EBR	CFRP strips	109.2	10.5	2	85–115	54–73	ISO834	85–580	72–180
				55	3.75	10					
				13.6	3.79	25					
[64]	1524 × 254 × 102	NSM EBR	CFRP tape			0	166	59.9–77.4	10–20	100	300 not fail *
										200	74.5

Not fail *: The specimen remained intact after 300 min of fire exposure, without reaching the failure criteria.

**Figure 9:**
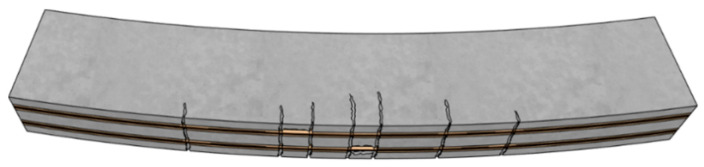
The post-fire observations of NSM-FRP strengthening beams.

**Figure 10:**
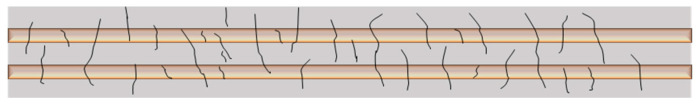
Failure mode of NSM-FRP strengthening beam.

Figure 9 presents the post-fire observations of NSM-FRP strengthening beams. The failure of the NSM-FRP reinforcement system at elevated temperature is mainly caused by the bond failure at the CFRP-adhesive interface. Due to the low temperature in the anchorage zone, the NSM-FRP strengthening system continues to maintain its strengthening effectiveness through the cable mechanism. However, as temperature increases, the slip of FRP composite increases and the mechanical properties of the reinforcement also decrease significantly, leading to the gradual increase in specimen deformation until its destruction. After exposure to elevated temperature, the inorganic adhesive containing fibers forms fine and dense cracks on the surface, as shown in Figure 10. This occurrence may be attributed to the melting of fibers within the inorganic adhesive, creating interconnected channels that allow water vapor to escape, effectively relieving internal stress [112].

At present, many studies suggest that the high temperature resistance of NSM FRP strengthening technique is better than that of EBR FRP strengthening technique. However, some studies indicate that the NSM strengthening specimen do not exhibit batter elevated temperature resistance than EBR strengthening specimen as anticipated. Paul et al. [64] performed experiments to investigate and compare the behavior of NSM- and EBR-strengthened concrete beam at moderate temperatures (100 °C and 200 °C). For NSM installations, the CFRP tape was bonded into grooves using commercial epoxy adhesive (T_g_ = 69 °C) and cementitious (unsanded silica fume) grout. It is worth noting that only half of the specimens were heated and the heating rate was approximately 10–20 °C/min (lower than the rate of the ISO 834 standard fire curve). Results indicated the cement-bonded NSM-FRP strengthening specimens G-100-1, G-200-1 and G-200-2 showed superior performance compared with epoxy-bonded NSM-FRP strengthening specimens. In particular, the G-100-1 specimen did not show signs of failure after heating at 100 °C for 5 h. The load then increased until failure occurred at 27.3 kN, which even displayed better performance compared with corresponding specimens loaded at ambient temperature. For the epoxy bonded NSM strengthened specimens, it lasted only about 40 min and 10 min at 100 °C and 200 °C, respectively. Specimen EB-100 bore a 20 kN load for more than four hours under 100 °C, and then the load was increased until failure occurred at 30.1 kN, representing 90% of the strength of corresponding specimens at ambient temperature. Furthermore, the specimen EB-200 showed surprising performance at 200 °C (approximately 140 °C higher than the T_g_ of epoxy adhesive), which was superior to specimens G-200-1 and G-200-2. The paper [64] attributes this phenomenon to the large bonding area of the EBR reinforcement method, necessitating a lower ratio of ambient temperature strength and stiffness to transfer stresses from the FRP to the concrete.

#### 3.1.4. Thermal Performance of FRP Confined Columns

In the field of fire safety research for FRP confined columns, the reinforcement of columns primarily relies on the EBR technique. By circumferentially wrapping FRP sheets or laminates around the column, the high tensile strength of the FRP provides lateral confinement to the core concrete, significantly enhancing both the axial load-carrying capacity and the ductility of the member [114,115]. As research has progressed, the focus has expanded from traditional organic bonding systems to inorganic cementitious systems with superior fire resistance, covering both “performance retention during fire” and “post-fire structural repair”.

For the widely used organic systems, Carbon FRP (CFRP) remains the dominant material due to its exceptional strength-to-weight ratio and high modulus [114,116]. However, the fire response of these systems is highly dependent on the thermal stability of the organic adhesive, typically epoxy resin. As previously mentioned, epoxy resins exhibit extreme sensitivity to temperature fluctuations. Studies consistently indicate that once the environment temperature exceeds the T_g_, the polymer matrix softens rapidly and loses its structural stiffness [115]. This physical transition leads to a breakdown in the stress transfer between the FRP and the concrete substrate, causing the reinforcement system to fail through interfacial debonding long before the fibers reach their ultimate tensile strength [114,115]. To mitigate this vulnerability, the application of fire insulation layers has proven highly effective. For instance, a 53 mm thick cementitious mortar insulation layer can significantly delay heat penetration, extending the fire endurance of a CFRP-wrapped column from 210 min to over 300 min under standard fire conditions [114]. Additionally, specialized fireproof coatings, such as Sikacrete 213F, can reduce internal temperature rise by an average of 65% after one hour of fire exposure [117].

In contrast to organic systems, Fabric-Reinforced Cementitious Matrix (FRCM), or Textile Reinforced Mortar (TRM), has emerged as a robust inorganic alternative. By replacing epoxy with mineral mortar as the bonding matrix, these systems are inherently non-combustible and do not release toxic fumes at high temperatures. In an FRCM system, high-performance fibers (such as PBO fibers) are embedded within the inorganic matrix. Although the tensile strength of PBO fibers may drop to approximately 48% at 300 °C, the cementitious matrix acts as an effective thermal barrier, slowing the rate of heat transfer to the column core [117]. Critically, in these experimental evaluations, the columns were first strengthened with the FRCM system and allowed to cure before being subjected to high-temperature exposure. Experimental data show that even a single layer of PBO-FRCM can reduce concrete surface temperatures by up to 31.9% and double the load-carrying capacity during fire exposure compared to unstrengthened columns. However, these systems are not immune to extreme heat; at temperatures exceeding 600 °C, the mineral matrix may crack due to dehydration, and the micro-bond between the fibers and the mortar gradually deteriorates, leading to a rapid decline in confinement efficiency [118].

The fundamental mechanism of FRP-strengthened columns is passive confinement. As the concrete column expands laterally under axial compression, the circumferential FRP is tensioned, exerting a reciprocal transverse pressure that places the core concrete in a favorable triaxial stress state [5]. Under fire conditions, this confinement not only compensates for the loss of material strength caused by heating but also acts as a physical shield to suppress concrete spalling [119]. In post-fire repair scenarios, applying multiple layers of CFRP to columns previously damaged by ISO-834 standard fire can achieve remarkable strength recovery, with capacity increases ranging from 224% to 971%. Interestingly, the confinement efficiency of FRP often increases with the severity of fire damage, as heat-damaged concrete exhibits a greater tendency for lateral expansion, thereby activating the FRP confinement earlier and more effectively [116].

The degradation of these strengthening systems follows a distinct phased pattern. In the early stages of fire (below 200 °C), performance loss is primarily attributed to the softening of the organic interface. As temperatures rise to 300–400 °C, unprotected organic matrices undergo irreversible thermal degradation or combustion, accompanied by the mechanical weakening of the fibers themselves. Beyond 600 °C, both the physical properties of the fibers and the integrity of the inorganic mortars are severely compromised [118]. It is critical to note that due to permanent chemical changes in the concrete microstructure (such as C-S-H dehydration and the formation of micro-cracks), the initial axial stiffness of the member can rarely be fully restored, even if the ultimate strength is recovered through FRP repair. Typically, a permanent stiffness loss of 80% to 90% remains. Furthermore, excessive FRP layering can impair permeability, promoting a rapid rise in vapor pressure under heating and increasing the risk of explosive spalling beyond that observed in unstrengthened columns [119].

As summarized in Table 7, the adhesive plays a dual role in FRP-strengthened columns by ensuring interfacial stress transfer and providing thermal insulation. Organic adhesives, represented by epoxy resins, significantly enhance the ultimate strength of columns under ambient conditions—reaching 131.2% to 197.0% of the capacity of unstrengthened columns. However, due to their inherent thermal sensitivity, these adhesives require an additional insulation layer (such as cementitious mortar, ECC, or UHPC with thicknesses ranging from 20 to 53 mm) to maintain structural integrity at high temperatures. In contrast, inorganic cementitious matrices (e.g., PBO-FRCM systems) demonstrate superior fire-resistant synergy, maintaining an ultimate strain increase of 169.2% even after prolonged fire exposure. Furthermore, integrating adhesives with Near-Surface Mounted (NSM) techniques or high-performance jackets effectively improves both the residual load-bearing capacity and the ductility of fire-damaged columns.

**Table 7:** Summary of the application of different adhesives in FRP confined columns.

Ref	Dimension of Specimen d × *h* (mm)	Strengthen Technique	FRP Type	Adhesive Type	Thermal Insulation		Heating Rate (°C/min)	Heating Time (min)	Strengthened Ultimate Strength/ Unstrengthened Ultimate Strength (%)	Strengthened Ultimate Strain/ Unstrengthened Ultimate Strain (%)
					Type	Thickness (mm)				
[114]	400 × 3180	EBR	CFRP	Epoxy resin	cementitious matrix	53	ASTM E119	300	\	75
[115]	200 × 800	EBR	Bidirectional PBO	cementitious matrix	cementitious matrix	30	ASTM E119	130	\	169.2
[119]	242 × 900	EBR	CFRP	Epoxy resin	cementitious matrix	30	ASTM E119	300	175.8	147.6
[118]	100 × 200	EBR	Bidirectional jute and basalt fiber	Epoxy resin	\	\	10 °C/min	40 + 120 (held) (held)	131.0	159.5
	100 × 200	EBR	Bidirectional jute and basalt fiber	cementitious matrix	\	\	10 °C/min	40 + 120 (held)	160.1	167.7
[120]	200 × 600	EBR	CFRP	Epoxy resin	ECC	25	ISO-834	30	175.8	372.5
	200 × 600	EBR	CFRP	Epoxy resin	concrete	25	ISO-834	30	176.8	293.3
	200 × 600	EBR	CFRP	None	ECC	25	ISO-834	30	185.8	239.5
[121]	150 × 500	NSM	BFRP	Epoxy resin	UHPC	20	2.5 °C/min	200	131.2	88.0
	150 × 500	NSM	BFRP	Epoxy resin	UHPC	20	2.5 °C/min	300	140.9	86.1
[122]	200 × 800	EBR	Bidirectional PBO	cementitious matrix	cementitious matrix	40	ASTM E119	180	\	27
	200 × 800	EBR	Bidirectional PBO	cementitious matrix	intumescent paint	<3	ASTM E119	180	\	128.1

### 3.2. Fire Resistance Coatings

To enhance the fire performance of EBR FRP-strengthened members, two main categories of passive protection are commonly adopted in practice: spray-applied fire-resistive materials (SFRM) and board/mortar fire protection systems.

For SFRM, Shin et al. investigated RC piloti-type columns confined with CFRP stirrups, where 10 mm, 20 mm, and 30 mm thick layers of SFRM or cementitious spray mortar were applied over the FRP jackets. A coupled thermal–structural analysis, calibrated against 4 h tests under the ASTM E119 standard fire curve, was conducted to evaluate the effectiveness of these protection systems [123]. The results showed a pronounced reduction in temperature rise at the FRP level with increasing SFRM thickness. For the column protected with 30 mm SFRM, the maximum temperatures at the CFRP location after 1, 2, 3, and 4 h of fire exposure were approximately 127 °C, 212 °C, 292 °C, and 363 °C, respectively, while the corner reinforcing bars remained within a range of about 82–327 °C—well below the commonly adopted steel critical temperature of 593 °C. Using T_g_ = 71 °C as a threshold for the loss of effective CFRP confinement, the unprotected column reached this temperature at the CFRP level in only about 3.2 min. When SFRM thicknesses of 10, 20, and 30 mm were provided, the time to reach T_g_ increased to approximately 10, 19, and 32.4 min, respectively, indicating a substantial extension of the period during which the CFRP could contribute structurally. More importantly, for SFRM thicknesses of 20 mm and above, the CFRP ignition temperature (taken as 450 °C) was not reached within the 4 h exposure, and the reinforcement temperatures remained below 593 °C throughout the test. Under these protection levels, the axial load-bearing capacity of the columns decreased by only about 25–36% after 4 h of standard fire exposure, compared with a reduction of approximately 58% for unprotected columns [123]. These findings demonstrate that adequately designed spray-applied fire protection not only delays FRP degradation but also continues to protect the concrete and reinforcing steel after FRP softening or loss of effectiveness, enabling the member to maintain an acceptable load-bearing capacity over the required fire resistance period.

Board and mortar protection systems enhance fire resistance primarily by increasing the heat capacity and thermal stability of the protective layer, thereby providing more sustained thermal insulation to the externally bonded FRP. Typical systems employ gypsum boards, fiber-cement boards, autoclaved aerated concrete (AAC) panels, or proprietary fire protection mortars, arranged around the FRP to form a multilayer composite enclosure. In full-scale tests on EBR CFRP-strengthened RC beams, Turkowski et al. applied 50–150 mm of gypsum or calcium silicate boards to the beam soffit, combined with AAC blocks at the top to ensure three-sided fire exposure [124]. Even when the strengthened beam was designed at 100% utilization at ambient conditions (i.e., its fire resistance still depended significantly on the CFRP contribution), appropriately selected board thicknesses enabled fire resistance ratings in excess of 4 h. In all protected specimens, failure occurred when the adhesive temperature approached or exceeded T_g_, triggering CFRP debonding at a stage where the midspan deflection was close to the limiting value or exhibited a marked increase. This behavior confirms that, for members relying heavily on FRP, the thickness and continuity of the board/mortar enclosure are critical parameters governing the achievable fire resistance level [124].

Recent comparative studies on NSM versus EBR systems have further highlighted the importance of protection detailing. Firmo, Arruda and co-workers reported that, in unprotected NSM CFRP-strengthened beams, the contribution of CFRP reinforcement was essentially lost after approximately 18 min under the ISO 834 standard fire heating process [62]. However, when a thin insulating layer was applied along the beam and 50 mm-thick calcium silicate boards were added locally at the anchorage zones to promote a “cable mechanism”, the effective working time of the CFRP could be extended to about 114 min [125]. In optimized configurations, the adhesive temperature at the anchorage zone at the instant of CFRP debonding could reach 2.4–5.0 times the T_g_, while the FRP still contributed through a tension-cable action [125]. These results clearly indicate that local thickening of the protection and enhancing anchorage robustness in critical regions—such as plastic hinge zones, column bases, and beam–column joints—can substantially improve the overall fire resistance of FRP-strengthened members.

Construction details and defects in the fire protection layer often control the practical effectiveness of these systems. Numerical and experimental studies by Shin et al. showed that, under two-sided heating of piloti-type columns, corner reinforcing bars experienced significantly higher temperatures than bars located on the sides; in the presence of discontinuities or gaps in the protection at corners, beam–column joints, or construction joints, hot gases and flames can rapidly penetrate to the FRP and steel, resulting in local temperatures far above the average section temperature and causing premature attainment of T_g_ and the steel critical temperature [123]. In parallel, Haris et al. reviewed experimental evidence indicating that epoxy adhesives already exhibit noticeable deterioration at 60–70 °C and that their bond strength may drop to approximately 20–30% of the ambient value in the 100–120 °C range [126]. If, due to local defects in the protection, such temperatures are reached early at the FRP-concrete interface, debonding tends to occur in a brittle and localized manner, undermining the effective thickness and durability of the fire protection system as a whole.

From a design perspective, these findings suggest that the fire resistance of externally bonded FRP-strengthened members is governed less by the intrinsic high-temperature strength of the FRP itself than by the ability of the protection system to control the temperature at the FRP and adhesive interface. Adequate SFRM or board/mortar protection must keep this interface temperature below T_g_, or at least significantly delay reaching T_g_, while also maintaining concrete and reinforcing steel temperatures below their respective critical values over the target fire resistance period. Experimental and numerical studies consistently indicate that, when spray-applied fire protection with a thickness of 20–30 mm or board/mortar protection on the order of 50 mm is provided, and when protection is locally thickened in anchorage regions, the effective fire resistance of both EBR and NSM FRP systems can be upgraded from 10 min to 2 h or more. In some fully utilized members, ratings exceeding 4 h have been demonstrated under standard fire exposure. These results provide a solid experimental and analytical basis for the safe application of externally bonded FRP strengthening in structures with stringent fire resistance requirements, and they highlight promising directions for future research on FRP-concrete interface modification and optimization of fire protection detailing.

### 3.3. Summary

Based on the synthesis of experimental studies on both FRP-strengthened beams and FRP-confined columns at elevated temperatures, the following conclusions can be drawn: (1) Regardless of the strengthening technique (EBR or NSM) and adhesive type (organic or inorganic), fire insulation remains the most critical factor for ensuring the survival of FRP-reinforced systems. For FRP-confined columns, the insulation layer significantly delays heat penetration, preventing the premature loss of the lateral confinement mechanism]. (2) The influence of the fire protection layer on fire endurance is much greater than that of the strengthening technique or adhesive type; for instance, a 30–50 mm thick insulation layer can extend fire resistance from mere minutes to several hours, effectively safeguarding the FRP from reaching its critical failure temperature. (3) In beam applications, the insulation layer facilitates a “cable mechanism” by protecting anchorage zones, while in column applications, it prevents explosive spalling and maintains the triaxial stress state of the core concrete.

However, several contradictions and complexities persist in the current literature: (1) While most studies suggest that the NSM method offers superior fire resistance over EBR due to the additional concrete cover, certain studies present opposite conclusions, likely due to differences in bond-slip behavior and thermal expansion compatibility [64]. (2) Although inorganic binders (e.g., FRCM/TRM) are intrinsically non-combustible and offer better thermal stability than epoxy resins, some studies indicate that epoxy-based systems may exhibit better temporary high-temperature resistance under specific low-load conditions, creating a conflict with the general consensus that inorganic systems are superior for fire safety [113]. (3) For confined columns, the transition from organic to inorganic binders significantly improves fire integrity; however, the lower interfacial shear strength of inorganic mortars compared to epoxies may lead to unexpected brittle debonding if the FRP surface is not specially treated.

## 4. Conclusions

This review has examined the fire performance of FRP materials and FRP–concrete systems from the material scale to structural applications, with a particular focus on EBR and NSM strengthening techniques and the implementation of fire-protection systems. Based on the synthesized literature, the following conclusions are drawn:

(1) The fire resistance of FRP systems is fundamentally constrained by the low T_g_ of the polymer matrix and adhesive, typically below 100 °C. Consequently, the governing failure mode is the premature loss of interfacial bond rather than fiber rupture. While alternative binders, including specialty high-T_g_ resins (e.g., phenolic or cyanate esters) and inorganic matrices, offer improved thermal stability, they currently exhibit limitations in ductility, cost, or processing that prevent them from serving as standalone solutions for all engineering scenarios.
(2) The presence and integrity of fire insulation are the dominant factors determining the fire survival time of both EBR and NSM-strengthened members, overshadowing the inherent differences between the two strengthening configurations. Adequate insulation thickness (e.g., 20–50 mm) can extend fire resistance from tens of minutes to 2–4 h. Without such protection, the structural efficacy of the FRP-concrete interface—even in NSM systems with partial concrete cover—is typically compromised within a very short duration upon reaching critical temperatures.

## 5. Prospects

While fiber-reinforced polymer (FRP) composites demonstrate significant advantages in structural reinforcement, their fire performance remains constrained by critical limitations. First, current experimental methodologies often employ low load ratios and neglect the mechanical interactions between high- and low-temperature zones in real fire scenarios, limiting the accurate assessment of FRP failure mechanisms and structural collapse behavior. Second, conventional fire-protection systems (e.g., sprayed fire-resistive materials and insulation boards) rely on substantial thickness (20–50 mm) to achieve fire resistance, which introduces challenges in construction complexity, cost, and weight. Third, the irreversible thermal degradation of polymer matrices (e.g., epoxy resins) at moderate temperatures (100–200 °C) leads to severe bond deterioration at the FRP-concrete interface, with anchorage effectiveness nearly vanishing above 300 °C. This contrasts sharply with steel’s partial recovery of mechanical properties post-cooling. Furthermore, inconsistencies in experimental outcomes—such as conflicting assessments of EBR versus NSM techniques or epoxy versus inorganic adhesives—highlight the lack of standardized testing protocols and unified design criteria. To address these challenges, future research should prioritize the following directions:

(1) Develop high-thermal-stability matrices (e.g., high-T_g_ epoxies, hybrid organic-inorganic binders) and advanced surface treatments (e.g., nano-coatings, sand-coated textures) to enhance bond retention and mitigate thermal mismatch with concrete at elevated temperatures.
(2) Conduct high-load-ratio fire tests incorporating realistic constraint effects (e.g., thermal expansion, restraint-induced stresses) to simulate real-world structural behavior and establish failure criteria for FRP-concrete systems. It is imperative to capture the true creep behavior and failure precursors of FRP systems under service loads.
(3) Establish standardized testing protocols and performance-based design frameworks to resolve inconsistencies in existing studies, particularly regarding strengthening configurations, adhesive types, and interfacial mechanics.
(4) The development of thin, high-efficiency fire-protective coatings (e.g., intumescent materials, nanocomposite insulators) should be accelerated to achieve comparable or superior thermal insulation performance at reduced thicknesses, particularly by enhancing early-stage fire resistance to mitigate epoxy resin degradation during critical initial fire phases. Additionally, hybrid systems integrating coatings with lightweight insulating materials should be explored to balance cost-effectiveness, constructability, and fire endurance through synergistic performance optimization.
(5) Prioritize protection of critical zones (e.g., anchorage regions, beam-column joints) to promote FRP cable mechanisms and delay debonding. Concurrently, develop post-fire assessment guidelines addressing residual strength and repair strategies, as FRP lacks the recovery capacity of steel after cooling.
(6) Establish temperature-dependent constitutive models and bond-slip relationships to enable reliable numerical simulations of FRP–concrete systems under coupled thermal-mechanical loads. Machine learning-driven predictive tools could further optimize fire design parameters.

By addressing these gaps through interdisciplinary collaboration, FRP composites can achieve the reliability required for safe deployment in fire-exposed environments, unlocking their full potential in sustainable infrastructure applications.

## Figures and Tables

**Table 1:** Basic mechanical properties of different FRP composites [4,8].

Fiber Type	Tensile Strength (MPa)	Elastic Modulus (GPa)	Ultimate Elongation (%)
Carbon Fiber			
High-strength type	3500–4800	214–235	1.4–2.0
Ultra-high-strength type	3500–6000	214–235	1.5–2.3
High-modulus type	2500–3100	350–500	0.5–1.0
Ultra-high-modulus type	2100–2400	600–700	0.2–0.4
Glass Fiber			
E-type	1900–3000	70–80	2.5–4.0
S-type	3500–4800	80–90	4.4–5.2
Aramid Fiber			
Low-modulus type	3500–4100	70–80	4.3–5.0
High-modulus type	3500–4000	120–130	4.5–5.5
Basalt Fiber	3450–4900	88–91	1.5–3.2

**Table 2:** Summary of the mechanical behavior of FRP-strengthened beam exposed to elevated temperatures.

Ref.	Type of FRP Composites	Applications	Research Methods	Fire Behavior Results	Mechanical Properties Results
[62]	CFRP laminates	To upgrade flexural strength of concrete beam	Four-point bending test ISO 834 standard fire test [63]	Without protection system/Debonding at 23 min 25 mm/Debonding at 60–89 min 40 mm/Debonding at 137–167 min	
[64]	CFRP tape	Comparing method NSM and EBR Investigating the effect of different kinds adhesive	Four-point bending test Heating rate: 10–20 °C/min Predetermined temperature: 100 °C and 200 °C	Without protection system Debonding at 11–84 min	
[65]	CFRP sheet	To investigate effect of elevated temperature on mechanical behavior of FRP strengthened concrete	Four-point bending test Heating rate: 4–5 °C/min Target temperature: 80, 160, 240 °C		The flexural strength decreases by 18.5% to 34% after exposure to 160 °C and 240 °C
[66]	CFRP strip	To investigate fire of concrete T-beams strengthened with NSM method	Four-point bending test ASTM E119 [67]	The NSM is better than EB in respect of fire behavior Temperature in rebars remained below 300 °C throughout fire exposure (3 h) (25 mm thickness fire insulation)	When the high load is applied (65%), the deflection of beam remained below 10 mm after 2 h heating, and that of 25 mm for 3 h heating
[68]	CFRP sheet	To investigate the fire behavior of FRP-strengthened beam, and the variables including type of fire exposure, anchorage zone, insulation type, restraint conditions	Four-point bending test Eurocode 1 parametric fire ASTM E119 standard fire	The rebar’s temperature is below 300 °C expect B2 (early development of crack) The test data showed similar temperature tend under different fire exposure	The deflection of beam remained below 20 mm, after 3 h heating. Cooler anchorage zone can result in cable action, which can restrain the development of deflection.

**Table 3:** Summary of the effect of temperature on the bonding performance between FRP and concrete.

Ref	Bar Type	Diameter (mm)	Surface Configuration	Bond Length (mm)	Test Method	Temperature (°C)	F_u_ (kN)	*τ_max_* (MPa)
[79]	CFRP	10	Sand coating	100	Pull-out	23	26.2	8.3
						125	22.0	7.0
						250	14.8	4.7
						325	4.8	1.5
						375	2.7	0.9
	GFRP	10	Helically wrapped	100	Pull-out	23	6.3	2.0
						125	4.6	1.5
						250	3.2	1.0
						325	1.3	0.4
	BFRP	10	Helically wrapped	100	Pull-out	23	8.3	2.6
						125	6.5	2.1
						250	3.2	1.0
						325	1.7	0.6
[89]	CFRP	14	Sand coating	N/A	Pull-out	20	N/A	5.8
						50		7.0
						80		7.7
	CFRP	12	Ribbed	N/A	Pull-out	20	N/A	12.7
						50		10.8
						80		11.0
[88]	CFRP	12.7	Sand coating	63.5	Pull-out	5	N/A	15.4
						20		13.3
						40		11.6
						80		12.4
	CFRP	9.5	Ribbed	47.5	Pull-out	5	N/A	20.6
						20		18.6
						40		18.2
						80		12.5
	CFRP	9.5	Deformed and textured	47.5	Pull-out	5	N/A	8.5
						20		7.9
						40		7.4
						80		7.1
[90]	CFRP	12	Sand coating	48	Pull-out	20	34.9	23.6
						150	31.3	21.2
						250	20.7	13.9
[80]	GFRP	9	Sand coating	45	Pull-out	23	N/A	9.9
						100		8.2
						150		7.7
						200		7.7
						250		6.9
						300		6.2
						400		5.7
						500		4.5
						600		3.4
[91]	GFRP	8	Ribbed	40	Pull-out	20	8.9	7.2
						60	7.6	6.2
						100	6.5	5.2
						140	6.7	5.4
						200	5.0	4.0
						250	2.2	1.8
						300	2.1	1.7
	GFRP	12	Ribbed	60	Pull-out	20	32.8	12.2
						60	28.9	10.7
						100	21.4	7.9
						140	22.6	8.4
						170	19.1	7.1
						200	9.6	3.6
						250	4.8	1.8
	GFRP	12	Ribbed	60	Pull-out	20	52.9	19.0
						60	48.8	17.6
						100	42.1	15.2
						120	22.8	8.2
						140	14.1	5.1
						220	6.0	2.2
						300	3.0	1.1

## Data Availability

The raw data supporting the conclusions of this article will be made available by the authors on request.
